# Purification and Characterization of Novel Antioxidative Peptides From Duck Liver Protein Hydrolysate as Well as Their Cytoprotection Against Oxidative Stress in HepG2 Cells

**DOI:** 10.3389/fnut.2022.848289

**Published:** 2022-03-15

**Authors:** Jin Sun, Changyu Zhou, Jinxuan Cao, Jun He, Yangying Sun, Yali Dang, Daodong Pan, Qiang Xia

**Affiliations:** ^1^State Key Laboratory for Managing Biotic and Chemical Threats to the Quality and Safety of Agro-Products, Ningbo University, Ningbo, China; ^2^Key Laboratory of Animal Protein Food Processing Technology of Zhejiang Province, College of Food and Pharmaceutical Sciences, Ningbo University, Ningbo, China; ^3^National R&D Center for Freshwater Fish Processing, Jiangxi Normal University, Nanchang, China

**Keywords:** duck liver peptides, antioxidant activity, RP-HPLC, molecular docking, cytoprotective effect

## Abstract

This study aimed at mining antioxidant peptides derived from duck liver as a strategy for valorizing poultry byproducts utilization *via* the isolation and characterization of peptide molecules with great antioxidant potential and cytoprotective effects against hydrogen peroxide-induced oxidative stress. Six novel peptides, including GEHGDSSVPVWSGVN, HLDYYLGK, HLTPWIGK, DTYIRQPW, WDDMEKIWHH, and MYPGIAD were isolated and purified by Sephadex G-15 and reverse-phase high-performance liquid chromatography, followed by the identification with liquid chromatography-tandem mass spectrometry. Among the hydrolysates from different enzymes, the alcalase-originated peptides presented the strongest antioxidant capacity as revealed by DPPH and ABTS assays. The synthesized peptides were used to validate the antioxidant activities, identifying that DTYIRQPW and WDDMEKIWHH were the major antioxidative peptides capable of protecting HepG2 cells from H_2_O_2_-induced oxidative damage *via* stimulating antioxidant enzymes such as superoxide dismutase and catalase to eliminate free radicals and to decrease lipid peroxidation products. Molecular docking suggested that the antioxidative properties of the isolated peptides were related to the site and number of hydrogen bonds. This investigation indicated the great potential of duck liver protein hydrolysates as a base material for producing and developing dietary bioactive peptides.

## Introduction

There is a growing interest in isolating and identifying antioxidant peptides from natural sources in recent years (1, 2). Antioxidant peptides can be produced by fermentation and enzyme hydrolysis, generally presenting 2–20 amino acids (AAs) residues of which the sequence is encoded in the parental protein (3). Recent researches have indicated that meat processing by-products, such as duck skin (4), porcine liver (5), porcine cerebral (6), Fish by products (7), Yak bones collagen (8) and duck plasma (9), can be a good source from which to mine antioxidant peptides with great bioactivities.

As a duck meat processing by-product, duck liver (DLv) possesses a large proportion of nutritional components such as proteins, fats, sugars, vitamins, and minerals, which possibly showing great value through the application of suitable valorization technologies (10). Generally, large varieties of proteins are synthesized in liver, and for DLv protein content accounts for about 20% of the total weight (11). Therefore, DLv could be a potential source for mining bioactive peptides, considering the relevance of antioxidant activities of peptides to AA composition and sequence (12). Current green methodologies were used for the acquisition of antioxidative peptides mainly include microbial fermentation and enzyme hydrolysis, involving the employment of digestive enzymes, or plant and microbial protease. Enzymolysis preparation is considered as fast, safe, highly repeatable and environmentally friendly to generate antioxidative peptides in a controllable way. Thus, this method has been the most commonly used method for industrial preparation of peptides (13). However, the impact of enzymolysis on the yields and transformation efficiency of antioxidant peptides from parent proteins is highly dependent on the employed enzyme types and food matrices, and specifically, to our knowledge, no literature has examined the antioxidative capabilities of bioactive peptides prepared from DLv using different enzymes despite its great potential.

To develop antioxidant peptides efficiently, it is of great significance to explore the quantitative structure-activity relationship (QSAR) of antioxidant peptides and to elucidate their antioxidant mechanisms, such as the roles of the active sites and structure of antioxidant peptides. It has been found that antioxidant properties of hydrolysates are closely related to the variation in AA composition and sequence (14–16). Specific AAs classes, including nucleophilic sulfur-containing AAs (Cys and Met), aromatic AAs (Trp, Tyr, and Phe), and the AA with imidazole groups (His) have been identified as the active sites of antioxidant peptides (13, 17). Nevertheless, the lack of general QSAR mechanisms can be one of major reasons limiting the application of antioxidant peptides at industrial scale, while traditional characterization of amino acid composition, sequences, and activity of peptides to establish QSAR tends to be time-consuming and costly. As an important complementary strategy, molecular docking modeling can be developed and applied to recognize key structural characteristics and QSAR between ligands and bioactive peptides (18). For example, QSAR models have been successfully developed to predict inhibitory activities of competitive inhibitors of dipeptidyl peptidase IV (DPP-IV), as revealed by the consistent ranking between the model-predicted and experimental results (19). In case of finger millet protein hydrolysate, molecular docking models were also established to reveal the interaction mechanisms of antioxidant peptide residues with free radicals (20).

The main purpose of this investigation was to separate, identify and characterize the antioxidant peptides from enzymatic hydrolysates of DLv protein. The extracted DLv protein was hydrolyzed by different enzymes, and the antioxidant capabilities from different hydrolytic fractions were compared. The major peptides responsible for antioxidative activities were further isolated and identified. Then, the antioxidative potential of the chemically synthesized peptides with the same sequences was evaluated by free radicals scavenging methods, and their reaction mechanisms and the possible binding sites were also discussed through establishing molecular docking simulation. Finally, the antioxidative effects were confirmed based on the protective effects against hydrogen peroxide-induced damage in HepG2 cells.

## Materials and Methods

### Materials

Fresh DLv samples were purchased from Beilun Food Co., Ltd. (Ningbo, China). Alcalase (200 U/mg), papain (800 U/mg), neutrase (60 U/mg), 1,1-diphenyl-2-picrylhydrazyl (DPPH), 2,2′-azinobis-(3-ethyl-benzothiazoline-6-sulphonate) (ABTS), and acetonitrile (HPLC grade) were purchased from Sigma (Beijing, China). Glutathione (GSH) and bicinchoninic acid (BCA) Protein Assay Kit were purchased from Sangon Biotech (Shanghai, China). Dulbecco's modified Eagle's medium (DMEM) was purchased from Gibco BRL Life Technology (Gibco BRL, Gaithersburg, MD, USA). Malondialdehyde (MDA), catalase (CAT), and superoxide dismutase (SOD) level assay kits were obtained from Nanjing Jiancheng Bioengineering Institute (Nanjing, China). All other reagents used were of analytical grade.

### Screening of Hydrolytic Enzymes for Preparing Antioxidative Peptides

DLv samples were soaked with water (1:6) and homogenized fully at 8,500 × g (DY89-II, Scientz Co., Ningbo, China). Afterwards, 1 mol/L sodium hydroxide was used to adjust the pH of solution to 12 and to stir constantly. Then the solution was centrifuged at 8,500 × g for 10 min, with the middle tier collected. The pH was then adjusted to 4.5 using 1 mol/L hydrochloric acid, and stirred for 10 min solution following with centrifuge (8,500 × g for 10 min, 4°C) (21). The precipitate was lyophilized and stored at −20°C until being used.

DLv protein was hydrolyzed using alcalase [pH 8, 50°C, E/S ratio (w/w) 1:100], neutrase [pH 7, 50°C, E/S ratio (w/w) 1:100], and papain [pH 7, 50°C, E/S ratio (w/w) 1:100]. The effects of enzyme hydrolysis were evaluated by antioxidant index and trichloroacetic acid-nitrogen soluble index (TCA-NSI) values. For the optimum hydrolysis process, the protein was suspended in distilled water (10% w/v) and the hydrolysis reaction was kept for 5 h under 50°C and suitable pH hydrolysis conditions for each enzyme. Thereafter, the mixture was boiled for 10 min for inactivating enzymes, followed by a centrifugation process at 8,500 × g, 4°C for 15 min (Eppendorf AG, Hamburg, Germany). The supernatants were lyophilized to use for further analysis.

### Antioxidant Activity Evaluation

The DPPH radical scavenging activity of the hydrolysates was determined according to Xing et al. (22). Briefly, the samples were blended with 1 mL of DPPH (0.2 M), with distilled water as the blank group. The solution was mixed with a vortex mixer and kept for 30 min in darkness before measuring the absorbance at 517 nm (model INFINTE 200PRO, Molecular Devices, Hombrechtikon, Switzerland). The radical-scavenging activity of the samples was compared with that of GSH. The formulation below was employed to calculate the DPPH radical-scavenging activity:


$$\begin{array}{l} \text{Scavengin activity }(\%)\;=\;[1\;-\;(\text{A}(\text{sample})\\ \;-\text{A}(\text{blank}))/(\text{A}(\text{control})\;-\text{ A}(\text{blank}))]\times \;100 \end{array}$$

ABTS radical scavenging activity was assessed following the method of Joshi et al. (23). At first, the equivalent volume of ABTS (7 mM) and potassium persulfate solution (2.45 mM) were mixed. Then, the solution was placed for 12 h at 25°C to generate ABTS+ under dark condition. The ABTS+ was diluted by phosphate buffer as a work solution to accomplish an absorbance of 0.7 ± 0.02 at 734 nm. The work solution (150 μL) and the hydrolysates (50 μL) were mixed, followed by incubation for 30 min. At last, the absorbance was determined at 734 nm, and the activity was calculated using the following equation:


$$\begin{array}{l} \text{ABTS scavenging ability }(\%)\;=\;(\text{A}(\text{control})\\ \;-\text{ A}(\text{sample}))/(\text{A}(\text{control}))\times \text{100} \end{array}$$

The hydroxyl radical scavenging activity was assayed, as previously described by Ren et al. (24). The experiment was carried out in a tube by mixing 0.1 mL of 10 mM FeSO4, 0.1 mL of 10 mM EDTA, 0.5 mL of 10 mM a-deoxyribose, 0.9 mL of sodium phosphate buffer (pH 7.4) and 0.2 mL of sample, followed by a gentle shaking step. The hydrogen peroxide (0.2 ml, 10 mM) was added to the mixture and incubated at 37°C for 60 min. After the test tubes were added with 1 mL of trichloroacetic acid (2.8%, TCA) and 1.0 ml of thiobarbituric acid (1.0%, TBA), the reaction of the mixture was carried out by heating on boiling water bath for 15 min. Then, the mixture was cooled down to room temperature. The absorbance at 532 nm was recorded, which was used to calculate the scavenging activity using the following equation:


$$\begin{array}{l} Hydroxyl\;scavenging\;ability\;(\%)\;=\;(\text{A}(\text{blank})\\ -\text{ A}(\text{sample}))/(\text{A}(\text{blank}))\times \;100 \end{array}$$

### Determination of TCA-NSI Value

The TCA-NSI value was measured using the method of Jang et al. (25) with few modifications. The supernatant from enzyme hydrolysis was blended with the equivalent volume of 20% of TCA to obtain 10% of TCA-soluble protein, after 1 h the mixture was centrifuged at 4,000 × g, 4°C for 15 min and readjustment of the pH. Subsequently, the collected supernatant was used to measure the protein content using the BCA method (26).


$$\begin{array}{l} \text{TCA }-\text{ NSI}(\%)\;=\frac{\text{TCA }-\text{ soluble protein}}{\text{Total protein}}\times \;100 \end{array}$$

### Peptide Purification by G-15 Chromatography and RP-HPLC

The Sephadex G-15 column (1.6 × 90 cm) was used to fractionate the crude extracts (1 mL, 10.0 mg/mL) and ultrapure water was used as the mobile phase (27). The flow rate was set at 1.5 mL/min, and the peptides were detected at 220 nm in the AKTA avant-150. Every elution peak was collected, of which the antioxidant activity was evaluated.

A high-performance liquid chromatography (HPLC) system (Agilent 1260 HPLC system, Agilent Technologies, CA, USA) equipped with a Symmetry C_18_ column (250 × 9.4 mm, 5 μm) was used to further separate the fractions with high antioxidative potentials. The mobile phases included 0.1% trifluoroacetic acetic acid (TFA) added with 5% acetonitrile (A) and acetonitrile (B). The linear gradient elution was carried out as follows: 0–30% in 15 min (mobile phase A), with a flow rate of 1 mL/min. The detection wavelength was set at 280 nm (28). The fractions were collected with an automatic collector for further identification.

### Peptide Sequence Identification by LC-MS/MS

The collected fractions were analyzed using nano-liquid chromatography-tandem mass spectrometry (LC-MS/MS; ThermoFisher Q Exactive system, ThermoFisher, USA), equipped with a C18 analytical column (Acclaim PepMap RSLC, 75 μm × 25 cm C18-2 μm 100 Å). Gradient elution was performed as follows: 0–30 min, 5–38% B (0.1%formic acid, 80% acetonitrile). The spray voltage and the ion transfer tube temperature were set at 1.9 kV and 275°C, respectively. The raw spectrum file collected by mass spectrometry was processed using PEAKS Studio 8.5 software (Bioinformatics Solutions Inc., Waterloo, Canada).

### Synthesis of the Isolated Peptides

To evaluate the antioxidant capability of the sequences, synthetic antioxidant peptides with the same sequences were synthesized chemically (Sangon Biotech, Shanghai, China), with the purities exceeding 98%. The DPPH and ABTS radicals-scavenging abilities of the synthetic peptides were compared through individually calculating the half-maximal inhibitory concentration (IC_50_) of the peptides.

### Molecular Docking of Antioxidant Peptides

The interaction process between the identified peptides and the free radicals including DPPH and ABTS were simulated with the AutoDock Tools software (http://autodock.scripps.edu/) according to Baba et al. (29) and Morris et al. (30) previously described with some modifications. The structures of DLv peptide were predicted by PEP-FOLD tool V3.5 (31). The pre-processing of peptides and ligand molecules was done by annotating atom types, partial charges, and hydrogen atoms. Ligand rigid root can be generated automatically and rotated. AutoDock 4.2 program and Lamarckian genetic algorithm were applied to determine the binding sites between the peptides and ligands. The conformers produced by a single docking simulation were selected on the basis of the lowest binding energy between peptides and ligands (20). The docking results were exported in the form of “pdbqt” files, and the graphics were generated and processed in PyMOL software (PyMOL software, version 2.4, San Carlos, USA).

### Cytoprotective Effect on H_2_O_2_-Induced Cell Damage

HepG2 cells were seeded into a 96-well culture plate at a density of 1 × 10^5^ cells/mL, and the survival rate of each group of cells was assayed using the cell counting kit-8 (CCK-8). The H_2_O_2_-induced (600 μmol/L) oxidative stress model was established to evaluate the protective effect of peptides (1, 10, 100, 500 μmol/L) on HepG2 cells, including the control group (100 μL of cell suspension), the damage group (100 μL of H_2_O_2_ solution), the peptide-protected group (1, 10, 100, 500 μmol/L peptide solution) and the positive control group (1, 10, 100, 500 μmol/L glutathione solution). Cells were cultured for 24 h before H_2_O_2_ treatments (600 μmol/L), followed by the determination of cell viability.

### Determination of Intracellular Reactive Oxygen Species and Oxidation Indicators

The contents of reactive oxygen species (ROS) were assessed by DCFH-DA probes as previously described (32). The fluorescence of HepG2 cells was observed by an inverted fluorescence microscope (Olympus IX53, Tokyo, Japan), and the fluorescence intensity was measured at the excitation wavelength of 485 nm and the emission wavelength of 525 nm with a microplate reader.

The activities of antioxidant enzymes were determined by the assay kits including superoxide dismutase (SOD) and catalase (CAT), as well as malondialdehyde (MDA), and the results of SOD, CAT, MDA expressed as U/mg prot and nmol/mg prot, respectively.

### Statistical Analysis

Data were expressed as means ± standard deviations of three experiments (the cell experiment was repeated six times). The statistical analysis was performed using SPSS 25.0 software (SPSS Inc., Chicago, IL, USA) and one-way ANOVA were applied to analyze the significance of differences in the results, with a significance level of *p* < 0.05.

## Results and Discussion

### Characterization of Enzymatic DLv Protein Hydrolysates

**Figure 1:**
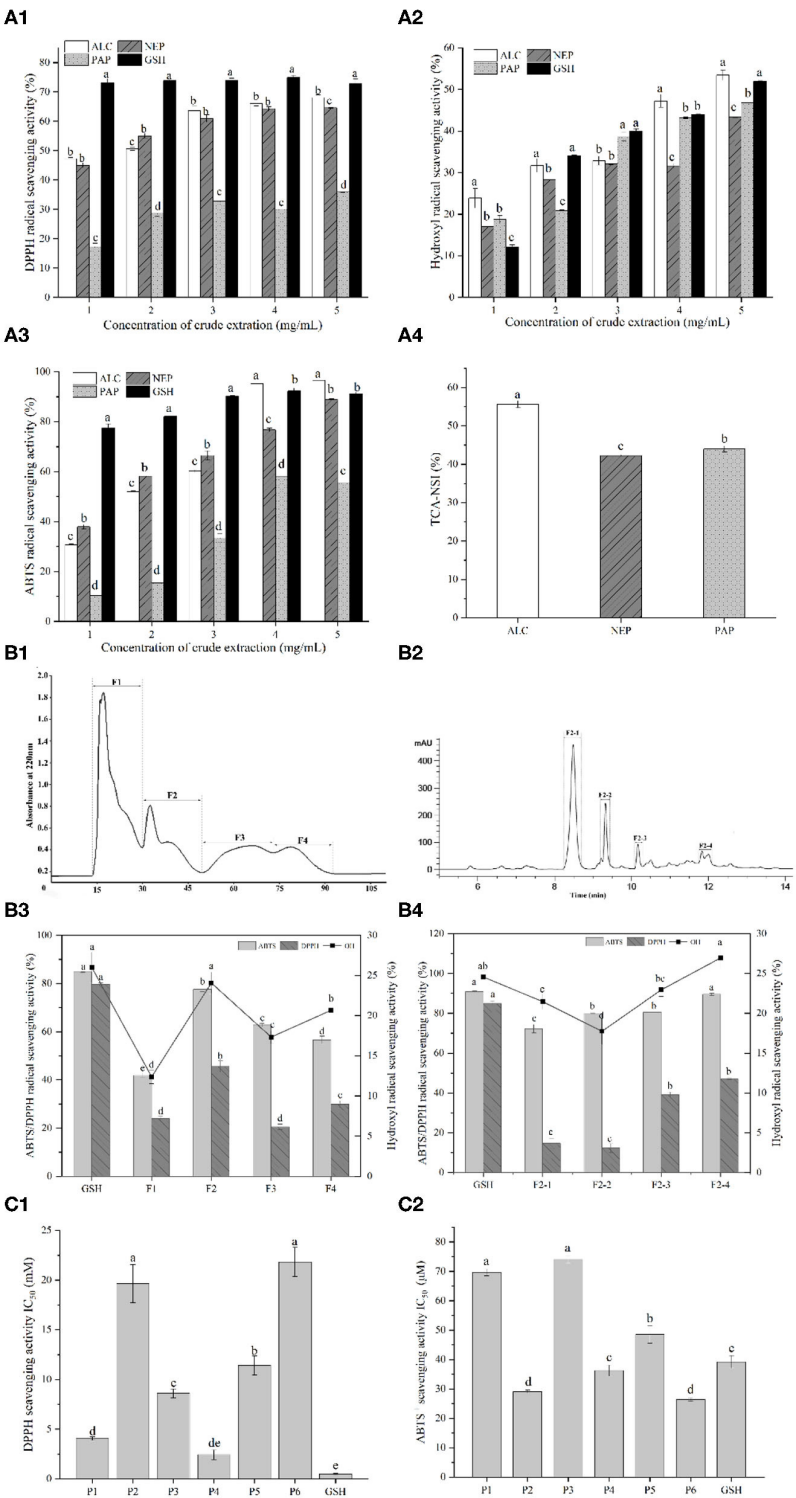
**(A1)** DPPH radical scavenging activity of duck liver protein hydrolysates prepared with alcalase (ALC), neutrase (NEP), and papain (PAP); **(A2)** Hydroxyl radical scavenging activity of different concentrations of ALC, NEP and PAP; **(A3)** ABTS radical scavenging activity of different concentrations of ALC, NEP and PAP. The synthetic glutathione (GSH) was used as the positive control. **(A4)** Trichloroacetic acid-nitrogen soluble index (TCA-NSI) of ALC, NEP and PAP derived hydrolysates. Different letters in the same group indicate significant differences according to Duncan's multiple range test (*p* < 0.05). **(B1)** Chromatogram profiles of duck liver peptides by gel filtration chromatography; **(B2)** chromatogram profiles of duck liver peptides by RP-HPLC; **(B3)** antioxidant activities of the subfractions of duck liver peptides after gel filtration chromatography; **(B4)** antioxidant activities of the subfractions of duck liver peptides after RP-HPLC. Different letters indicate significant differences by Duncan's multiple range test (*p* < 0.05). The DPPH radical scavenging activity **(C1)** and ABTS scavenging capability **(C2)** of the synthesized antioxidant peptides. P1, GEHGDSSVPVWSGVN; P2, HLDYYLGK; P3, HLTPWIGK; P4, DTYIRQPW; P5, WDDMEKIWHH; P6, MYPGIAD.

The antioxidant activities of the different hydrolysates were determined using DPPH, ABTS and hydroxyl radical scavenging assays (Figure 1A). In the concentration range of 1 to 5 mg/mL, the antioxidant capabilities of all the hydrolysates were increased gradually. Regarding the effects of enzyme types on antioxidant activities, alcalase-derived hydrolysate showed the highest ABTS^+^ and hydroxyl radical scavenging rates among all the enzymes. Alcalase even led to a higher ABTS scavenging capability than GSH in the range of 4–5 mg/mL, suggesting that the action mode of alcalase on peptide bonds favored the higher yields of antioxidative peptides compared with papain and neutrase.

Alcalase is a non-specific protein endopeptidase of microbial origin, and it mainly acts on the carboxy-terminal peptide bonds of Glu, Met, Leu, Tyr, Lys, and Gln. In the cases of bullfrog skin protein, it was also found that alcalase-derived hydrolysates demonstrated the highest antioxidant activities among all the enzyme hydrolysates catalyzed by neutrase, pepsin, papain, α-chymotrypsin and trypsin (17). Similar results from Ren et al. (24) have also showed that hydrolyzing grass carp muscle with alcalase presented the antioxidant properties. Therefore, from the perspective of the *in vitro* antioxidant potential, it was speculated that alcalase-catalyzed hydrolysis may result in more abundant forms of antioxidant peptides, including shorter peptides and diversified biological activities. This phenomenon could be confirmed by nitrogen solubility index as it is directly related to the contents of low-molecular-weight oligopeptides and free AAs (33). In fact, TCA-NSI value can be used as a reference indicator for the hydrolysis capacity, inferring the degree of hydrolysis of DLv protein. The highest TCA-NSI value (55.62 ± 0.89%) was found for alcalase-derived hydrolysates, followed by the papain (43.98 ± 0.79%) and neutrase (42.24 ± 0.21%) (Figure 1A4), suggesting that the alcalase hydrolysates attained a higher oligopeptide content than other hydrolytic enzymes. These results demonstrated that alcalase-derived DLv protein hydrolysates could be potential antioxidant peptides with great bioactivities which were further isolated and characterized.

### Peptide Purification by G-15 Chromatography and RP-HPLC

To purify and identify the antioxidative peptides, DLv protein hydrolysates were subsequently applied to gel filtration chromatography and RP-HPLC system. Four sub-fractions (F1, F2, F3, F4) were obtained from alcalase-derived hydrolysates by Sephadex G-15 (Figures 1B1,B2). Among all the pooled and lyophilized fractions, the fraction F2 at 1 mg/mL was found to simultaneously exhibit the highest DPPH radical scavenging activity (45.55 ± 2.37%), ABTS radical scavenging activity (77.68 ± 1.08%), and hydroxyl radical scavenging activity (24.06 ± 1.33%) (Figure 1B3). Therefore, the fraction F2 was further separated by RP-HPLC, further fractionated into four portions. As shown in Figure 1B4, among all fractions, fraction F2-4 possessed the highest hydroxyl radical scavenging activity (26.95 ± 1.30%), followed by the fractions F2-3 (22.94 ± 0.82%), F2-1 (21.48 ± 0.98%), F2-2 (17.74 ± 1.64%), and even higher than GSH group (1 mg/mL, 24.56 ± 0.78%). Particularly, DPPH, ABTS and hydroxyl radical scavenging activities of F2-4 were significantly higher (*p* < 0.05) than the crude fractions (F1, F2, F3, F4). The increased antioxidative activities after subfractionation by Sephadex gel filtration and HPLC systems were due to the efficient enrichment and purification of the bioactive peptides with antioxidative potential by the combined chromatographic separation technologies. Similar phenomenon has been observed in multiple protein hydrolysates, including antioxidative peptides from the protein hydrolysates of rapeseed, loach and pearl millet (21, 34, 35).

### Identification of Antioxidant Peptides by LC-MS/MS

The fraction with the highest antioxidant activity was further isolated and identified using LC-MS/MS. Six novel peptides, including GEHGDSSVPVWSGVN (P1), HLDYYLGK (P2), HLTPWIGK (P3), DTYIRQPW (P4), WDDMEKIWHH (P5), and MYPGIAD (P6), were identified. These peptides have 7–15 AAs and the molecular masses of 765.33–1,525.68 Da. Previous reports have demonstrated that most of the antioxidant peptides from food protein have molecular weights of 500–1,800 Da. Ranathunga et al. (36) also reported that low-molecular-weight peptides showed relatively high antioxidant activities, related to the steric hindrance and the enhanced interaction between peptides and free radicals.

The peptides identified above were further synthesized to verify their antioxidant activities, as shown in Figure 1C, where it can be seen that these peptides showed significant differences in the antioxidant activity depending on the types of peptides. Particularly, the ABTS radicals-scavenging activities of HLDYYLGK (P2), DTYIRQPW (P4), and MYPGIAD (P6) exceeded that of the positive control GSH, as revealed by the lower IC_50_ values than GSH group, with being 29.13 ± 0.49 μM, 36.23 ± 1.86 μM, and 26.40 ± 0.59 μM, respectively. The existence of Tyr residue in these peptides was observed, which was reported to have a relationship with the strong ability to remove ABTS^+^, due to the hydrogen/electron-donating ability of the residue (37). The presence of acidic AAs residues at the N-terminus, such as Asp, may be also responsible for the antioxidant properties of the DTYIRQPW (P4). It was also found that for acidic AA residues at higher pH (e.g., Asp or Glu), their carboxyl residues were deprotonated to form anions which thus exhibited a strong antioxidant capacity to suppress the lipid peroxidation (38). Besides, His has been reported to represent strong radical-scavenging activity due to the decomposition of its imidazole ring (38, 39). This residue was found in GEHGDSSVPVWSGVN (P1), HLDYYLGK (P2), HLTPWIGK (P3), and WDDMEKIWHH (P5), possibly contributing to their antioxidant properties. These results further confirmed that the antioxidant properties of peptides were not only influenced by the distribution of molecular weight, but also by the sequence and physicochemical properties, such as amphiphilic nature, of AAs in the chain (21).

### Molecular Docking Simulation

**Figure 2:**
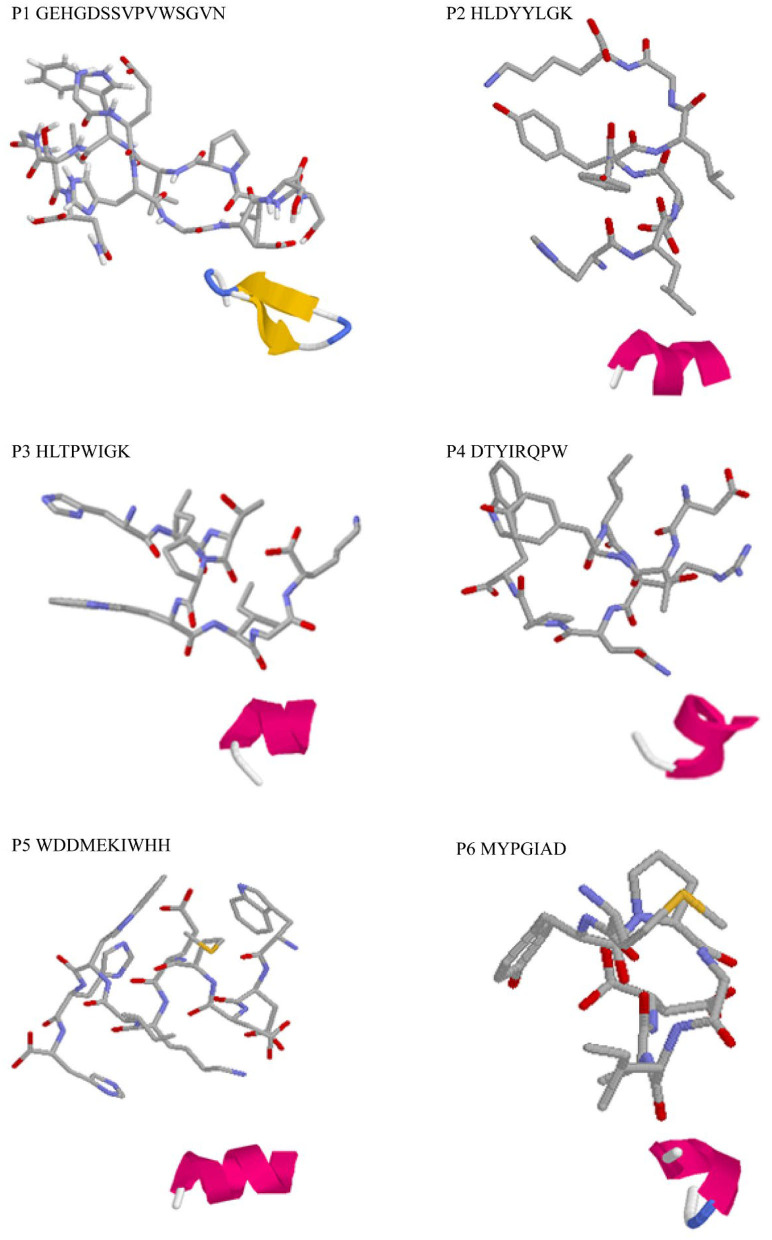
3D structure prediction of the identified antioxidant peptides. Each master figure showed the 3D modeling of the identified peptide molecule, while the attached figure shows the folding pattern of the molecule in the aqueous solution: green, β-turn; blue, β-sheet; red, α-helix; gray, random coil.

Three-dimensional structure predictions indicated that all identified peptides have α-helix structure except P1 (Figure 2). The secondary structures, particularly α-helix, β-turn, and β-sheet with net-charged and/or hydrophobic residues were demonstrated to play key roles in determining antioxidant capacity of peptides and could be used as efficient indicator as high antioxidant activity (40). Simultaneously, it was confirmed that varied spatial conformation of the peptide also affected its biological activity (41). Therefore, to further investigate the antioxidant mechanism of peptides, the binding mode between DPPH, ABTS, and peptides was predicted by molecular docking.

**Figure 3:**
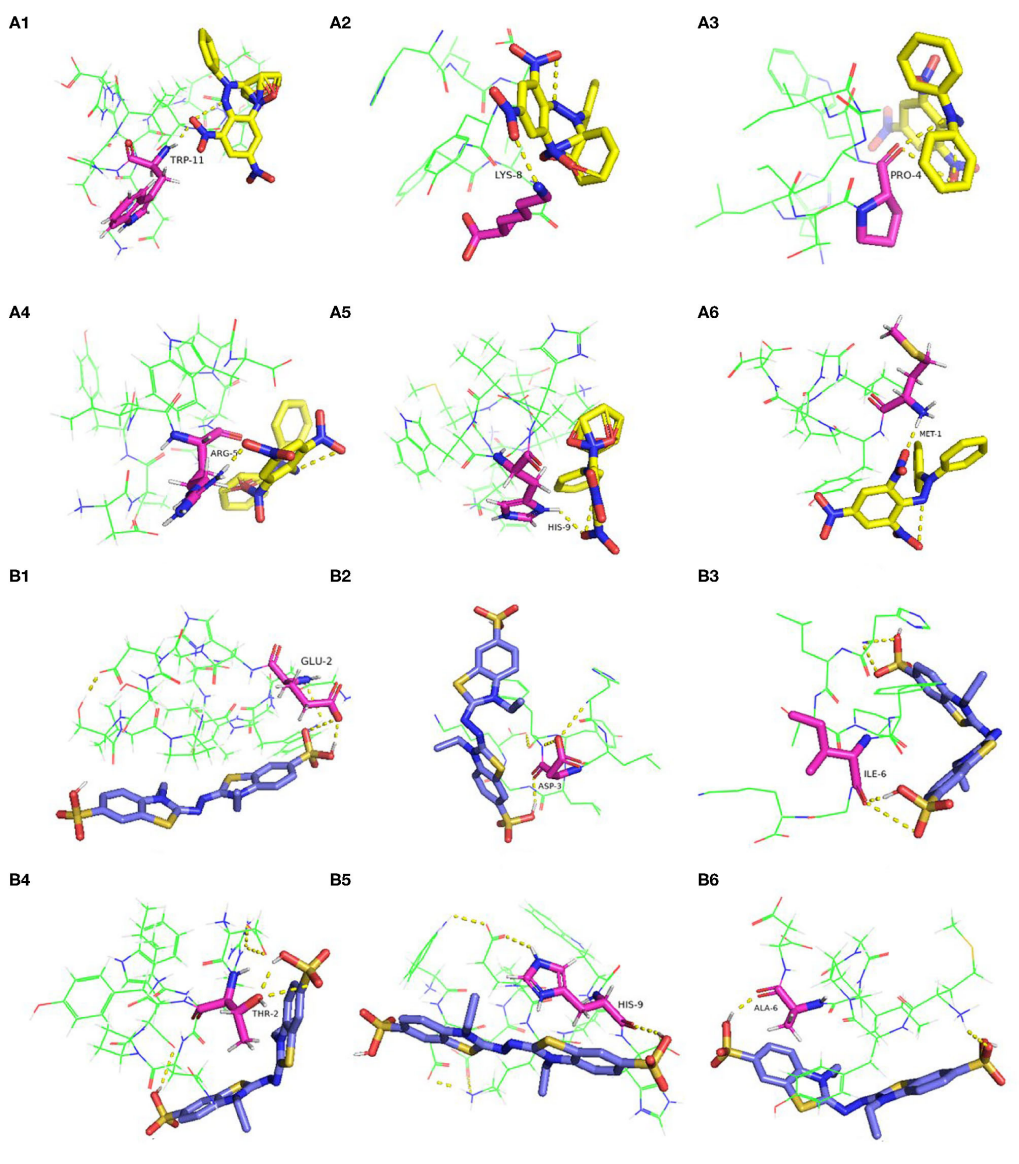
Molecular docking of the antioxidant peptides with DPPH. **(A1)** GEHGDSSVPVWSGVN; **(A2)** HLDYYLGK; **(A3)** HLTPWIGK; **(A4)** DTYIRQPW; **(A5)** WDDMEKIWHH; **(A6)** MYPGIAD. Molecular docking of the antioxidant peptides with ABTS. **(B1)** GEHGDSSVPVWSGVN; **(B2)** HLDYYLGK; **(B3)** HLTPWIGK; **(B4)** DTYIRQPW; **(B5)** WDDMEKIWHH; **(B6)** MYPGIAD. The yellow, blue and pink represent DPPH, ABTS and amino acid residues of the peptide, respectively.

Researches have shown that the peptides often interact with free radicals through hydrogen bonds, hydrophobic, van der waals, and electrostatic interactions (42, 43). In this work, the molecular docking results revealed that hydrogen bonds were formed between the antioxidative peptides and free radicals. According to Figure 3A, the active centers of peptides (P1–P6), such as TRP-11, LYS-8, PRO-4, ARG-5, HIS-9, and MET-1, interacted with DPPH to form hydrogen bonds with an average bond length of 2.19 Å. Zheng et al. (44) confirmed that hydrophobic AAs such as Trp, Met, and Ala played a dominant role in antioxidant peptides, which were the major AAs responsible for the binding sites of antioxidant peptides with DPPH or ABTS free radicals (Figure 3). Two hydrogen bonds were formed between HLTPWIGK (P3)/DTYIRQPW (P4) and DPPH, presenting higher rates of scavenging DPPH free radicals than other peptides forming a single hydrophobic bond. Mirzaei et al. (43) confirmed that the hydrogen bond interaction force contributed to stabilize the docking complex and catalytic reactions. P1-P6 formed hydrogen bonds with ABTS on AAs residues such as GLU-2, ASP-3, ILE-6, THR-2, HIS-9, ALA-6, and the average bond length was 2.17 Å. As shown in Figure 3B, two hydrogen bonds were formed between DTYIRQPW (P4)/MYPGIAD (P6) and ABTS. Thus, it could be concluded that AAs residues in peptides can effectively combine with free radicals by forming hydrogen bonds, thus resulting in strong free radical scavenging ability.

### Cytoprotective Effects Against H_2_O_2_-Induced Oxidative Damage

**Figure 4:**
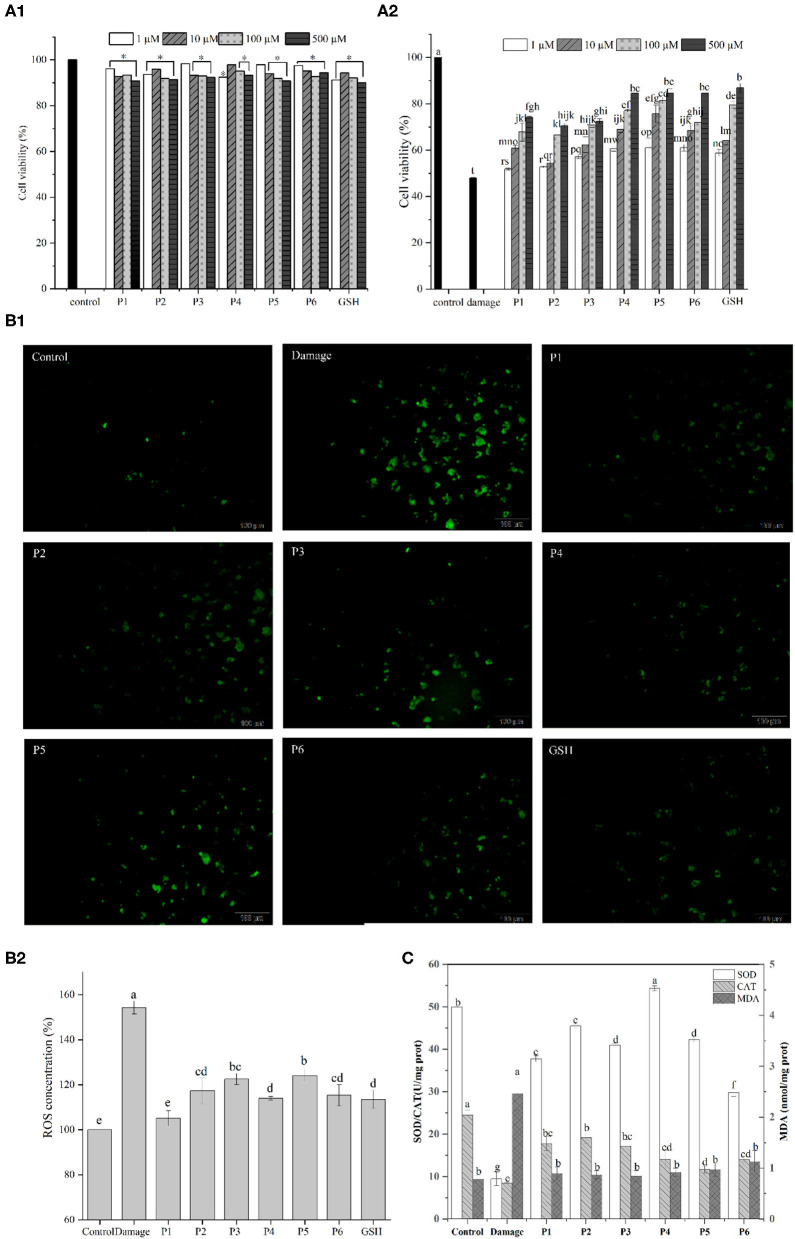
**(A1)** Effect of the antioxidant peptides on the survival rates of HepG2 cells. HepG2 cells were incubated with peptides at nominal concentrations (1, 10, 100, and 500 μm /L) for 24 h. **(A2)** Protective effect of synthesized antioxidant peptides to against H_2_O_2_-induced stress damage in HepG2 cells. **(B)** Effects of DLv antioxidant peptides (100 μmol/L) on ROS levels of H_2_O_2_-stressed HepG2 cells. **(C)** Effects of DLv-derived antioxidant peptides on the activities of SOD and CAT as well as the MDA content in H_2_O_2_-induced HepG2 cell.

The effects of duck liver antioxidant peptides on the viability of HepG2 cells are shown in Figure 4A. The cell viability of all groups supplemented with antioxidative peptides were >90% compared to the control group, indicating that the isolated peptides were not toxic to the cells. Hydrogen peroxide easily penetrates cell membranes and stimulates cells to produce oxidative stress, thus inducing cell apoptosis (45). Compared with the control group, the cell viability of damaged group treated with H_2_O_2_ was 47.99%. Except for P1 group (1 μM), the cell viability of other groups increased significantly compared to the damaged group. Particularly, the cell survival rate of P5 group (500 μM) was 1.76 times higher than that of the damaged group. The results showed that the antioxidant peptides contributed to improve the survival rates of H_2_O_2_-induced injured cells in a dose-dependent way, consistent with the recently reported results (46, 47). Therefore, it is concluded that these antioxidative peptides present significant cytoprotective effects against the hydrogen peroxide-induced oxidative damage in HepG2 cells.

Excessive reactive oxygen can cause oxidative damage and dysregulation of normal metabolism and physiology in cell and tissue (48). According to Figure 4B, compared with the control group, the ROS level in damaged group was significantly increased to 153.31 ± 2.84% which demonstrated enhanced oxidative stress on cells after H_2_O_2_ treatment. The addition of antioxidative peptides, along with GSH, led to the significantly decreased fluorescence intensity, while the intracellular ROS levels in the positive control group was lower than that of the groups supplemented with the peptides. It was noted that the levels of ROS in the GSH group were greatly higher (*p* < 0.05) than P1 group (105.04 ± 3.41%), but no significant difference was observed (*p* ≥ 0.05) between GSH group and the groups supplemented with the peptides of P2, P4, P6. Therefore, DLv-derived antioxidative peptides can reduce the production of intracellular ROS and in turn the decreased oxidative damage by eliminating free radicals.

To further elucidate the cytoprotective effects of DLv peptides on H_2_O_2_-damaged cells, intracellular MDA levels and the activities of antioxidant enzymes including SOD and CAT were determined. According to Figure 4C, SOD level in the control group was 49.98 ± 0.13 U/mg prot, and that of the H_2_O_2_-damaged group was reduced to 9.49 ± 1.60 U/mg prot. However, DLv-derived antioxidant peptides significantly improved the SOD levels in cells, among which the peptide P4 presented the highest levels and reached 54.35 ± 0.57 U/mg prot. Similarly, compared with the control group, CAT level in the damaged group was reduced prominently to 8.49 ± 0.12 U/mg prot. Pretreatment with antioxidant peptides (P1-6) significantly increased the activity of CAT which converts hydrogen peroxide into oxygen and water. P1-3 presented a more obvious impact on increasing the activity of CAT than the peptides of P4-6, suggesting the difference in mitigating oxidative damage in HepG2 cells among the peptides. It was noted that the ROS content of P1 group was significantly lower than that of P2, while the activities of SOD and CAT of P1 group were lower than those of P2, suggesting that there were other antioxidant enzyme systems and small molecules playing a key role in exerting antioxidant effects in cells, such as glutathione peroxidase and glutathione.

Intracellular ROS can attack polyunsaturated fatty acids in the biofilm and trigger lipid peroxidation to form MDA (49), of which the contents can reflect the change of the antioxidant system of cells to a certain extent. The damaged group obviously increased the MDA level in comparison with the control group (*P* < *0.05*). However, the pretreatment with antioxidant peptides significantly decreased MDA production as induced by H_2_O_2_ (*P* < *0.05*). Thus, it can be concluded that DLv antioxidant peptides contribute to protecting cells by reducing the oxidative stress injury due to the elevated activities of antioxidant enzymes (CAT, SOD), as well as the resultant decrease in the production of MDA and ROS. Similar results have been reported in the recently published literatures in which antioxidant peptides from shrimp and moringa oleifera seeds significantly decreased the level of intracellular ROS and upregulated the SOD activity (50, 51).

## Conclusions

In this study, six novel antioxidant peptides including GEHGDSSVPVWSGVN, HLDYYLGK, HLTPWIGK, DTYIRQPW, WDDMEKIWHH, and MYPGIAD were isolated and identified, for the first time, from DLv protein hydrolysates by alcalase enzyme using gel filtration chromatography, RP-HPLC and LC-MS/MS. The antioxidant capacities of the different hydrolysates were compared by evaluating their capability to scavenge the free radicals including DPPH, ABTS, and hydroxyl, among which the alcalase-derived hydrolysates presented the strongest antioxidant capacity. The results of antioxidant capabilities were further confirmed using the synthetic peptides, and molecular docking simulations were performed to characterize the interaction process between the antioxidative peptides and the free radicals, demonstrating that the formation of hydrogen bonds and the presence of hydrophobic AAs could be the major factors responsible for radicals-scavenging activities of antioxidative peptides. Furthermore, the intracellular antioxidant activities of the six antioxidant peptides were validated by examining their cytoprotective effects against H_2_O_2_-induced oxidative damage of HepG2 cells, mainly related to the enhanced activities of SOD and CAT as well as inhibition of lipid peroxidation. The results of this study indicated the potential feasibility of preparing natural antioxidant peptides from duck liver protein hydrolysates. Further studies concerning the bioavailability and protective mechanisms of antioxidative peptides under *in vivo* situations are to be prepared.

## Data Availability Statement

The original contributions presented in the study are included in the article/supplementary material, further inquiries can be directed to the corresponding author/s.

## Author Contributions

JS: investigation, formal analysis, and writing—original draft preparation. CZ: methodology. JH: data curation. YS: project administration. YD: validation. JC: supervision. QX: writing—reviewing and editing. DP: conceptualization and funding acquisition. All authors contributed to the article and approved the submitted version.

## Funding

This work was financially supported by the Department of Science and Technology of Zhejiang Province (2019C02085), National Key R&D Program of China (2021YFD2100104), the Department of Science and Technology of Ningbo City (2019C10017 and 202002N3076), and the Modern Agricultural Technical System Foundation (CARS-42-25).

## Conflict of Interest

The authors declare that the research was conducted in the absence of any commercial or financial relationships that could be construed as a potential conflict of interest.

## Publisher's Note

All claims expressed in this article are solely those of the authors and do not necessarily represent those of their affiliated organizations, or those of the publisher, the editors and the reviewers. Any product that may be evaluated in this article, or claim that may be made by its manufacturer, is not guaranteed or endorsed by the publisher.
